# Genetic variant of *PRKAA1* and gastric cancer risk in an eastern Chinese population

**DOI:** 10.18632/oncotarget.6124

**Published:** 2015-10-15

**Authors:** Li-Xin Qiu, Jing He, Lei Cheng, Fei Zhou, Meng-Yun Wang, Meng-Hong Sun, Xiao-Yan Zhou, Jin Li, Wei-Jian Guo, Ya-Nong Wang, Ya-Jun Yang, Jiu-Cun Wang, Li Jin, Xiao-Dong Zhu, Qing-Yi Wei

**Affiliations:** ^1^ Department of Medical Oncology, Fudan University Shanghai Cancer Center, Department of Oncology, Shanghai Medical College, Fudan University, Shanghai, China; ^2^ Cancer Institute, Collaborative Innovation Center for Cancer Medicine, Fudan University Shanghai Cancer Center, Shanghai, China; ^3^ Department of Pediatric Surgery, Guangzhou Women and Children's Medical Center, Guangzhou Medical University, Guangzhou, China; ^4^ Department of Pathology, Fudan University Shanghai Cancer Center, Shanghai, China; ^5^ Department of Gastric Cancer & Soft Tissue Sarcoma Surgery, Fudan University Shanghai Cancer Center, Shanghai, China; ^6^ Ministry of Education Key Laboratory of Contemporary Anthropology and State Key Laboratory of Genetic Engineering, School of Life Sciences, Fudan University, Shanghai, China; ^7^ Fudan-Taizhou Institute of Health Sciences, Jiangsu, China; ^8^ Duke Cancer Institute, Duke University Medical Center, Durham, NC, USA

**Keywords:** PRKA71, polymorphism, gastric cancer, genetic susceptibility

## Abstract

Published data on the association between *PRKAA1* rs13361707 T > C polymorphism and gastric cancer (GCa) susceptibility were inconclusive. To derive a more precise estimation of the association, we conducted a large-scale GCa study of 1,124 cases and 1,194 controls to confirm this association in an Eastern Chinese population. Our results showed that the C allele of *PRKAA1* rs13361707 increased the GC risk in the study population [CT vs. TT, odds ratio (OR) = 1.72, 95% confidence interval (CI) = 1.40–2.12; CC vs. TT, OR = 2.15, 95%CI = 1.70–2.71; CT/CC vs. TT, OR = 1.86, 95%CI = 1.53–2.26; CC vs.TT/CT, OR = 1.49, 95%CI = 1.24–1.79]. In addition, the association of C allele with an increased GCa risk was still significant in subgroups, when stratified by age, sex, tumor site, drinking and smoking status. Moreover, the findings in the present study were validated by our further meta-analysis. In summary, these results indicated that the C allele of *PRKAA1* rs13361707 was a low-penetrate risk factor for GCa.

## INTRODUCTION

Gastric cancer (GCa) is currently the most frequently occurring cancer and one of the leading causes of cancer-related death in the world. A total of 951,600 new GCa cases and 723,100 deaths are estimated to have occurred in 2012, accounting for 8% of the total cases and 10% of total deaths [1]. Although mechanism of gastric carcinogenesis is still not fully understood, it has been suggested that environmental factors combining with low-penetrance susceptibility genes may be important. For example, a high rate of *Helicobacter pylori* (*HP*) infection (70–90%) in developing countries (compared with 25–50% in developed countries) might be a potential risk factor for GCa [2, 3]. However, because only few *HP* carriers eventually develop GCa, other factors must play a role in GCa risk. Life styles, such as tobacco smoking and diet, are also suggested as potential risk factors for GCa [4], but the relevant data are limited. On the other hand, although studies have shown an association between high body mass index and GCa risk in developed countries [5], this association is weak in Chinese populations [6]. Up to now, genetic factors for GCa risk are still not fully recognized.

Recent studies have shown significant associations between genetic variants and GCa risk [7–10], but additional confirmation in different population is needed. For example, three GCa GWAS studies reported that SNPs in *PSCA, MUC1,* and *PLCE1* were associated with an increased GCa risk [11–13]. More recently, the rs13361707 SNP in *PRKAA1* (encoding protein kinase, AMP-activated, alpha 1 catalytic subunit) pathway was newly identified by a GWAS study in Chinese Han population as a risk factor for non-cardia GCa [14].

The rs13361707 SNP is located in the first intron of *PRKAA1* at 5p13.1. The PRKAA1 protein is one of the subunits of the mammalian 5′-AMP–activated protein kinase (AMPK), a central metabolic switch found in all eukaryotes that governs glucose and lipid metabolism in response to alterations in nutrient and intracellular energy levels [15]. AMPK has been implicated in a number of diseases related to energy metabolism, including cancer.

However, it was noted that Chinese populations included in these GWAS studies and replications were mostly northern (e.g., Beijing City) and southern Chinese (e.g., Nanjing City) populations, and the results in these reports were not always consistent. Of note, one case-control study did not support the association between rs13361707 SNP and GCa risk [16]. Moreover, some bias was considered inevitable in the published studies because of population stratification.

To fill these gaps, first, we conducted a replication study on the association between *PRKAA1* rs13361707 SNP and GCa risk in a large eastern Chinese population rarely included in previous studies. Second, to avoid regional bias, which may be caused by different genetic background, we carried out a meta-analysis to increase statistical power for assessing the association between *PRKAA1* rs13361707 SNP and GCa risk.

## RESULTS

Baseline information of the study population was similar to our previous study [17]. One sample in cases and two samples in controls failed to be genotyped. Thus, a total of 1,124 GCa patients and 1,194 cancer-free controls were included in the final analysis. Individuals were well matched by age and sex. The allele frequency of this SNP in control group was in line with HWE.

Table 1 listed the allele frequency of rs13361707 T > C SNP in cases and controls and the association of the rs13361707 T > C SNP with GCa risk. The results indicated that the C allele of *PRKAA1* rs13361707 increases GC risk[CT vs. TT, odds ratio (OR) = 1.72, 95% confidence interval (CI) = 1.40–2.12; CC vs. TT, OR = 2.15, 95%CI = 1.70–2.71; CT/CC vs. TT, OR = 1.86, 95%CI = 1.53–2.26; CC vs.TT/CT, OR = 1.49, 95%CI = 1.24–1.79; and additive model, OR = 1.46, 95%CI = 1.30–1.64] in the study population. The population attributable risk of CT, CC, CT/CC alleles were 0.53%, 11.51% and 4.4% respectively.

**Table 1:** Logistic Regression Analysis of Associations between the Genotypes of PRKAA1 rs13361707 T > C and Gastric Cancer Risk in an eastern Chinese Population

Genotype	Cases (*N* = 1,124)	Controls (*N* = 1,194)	Crude OR (95% CI)	*P*	Adjusted OR (95% CI) ^a^	*P* ^a^
TT	209 (18.6)	356 (29.8)	1.00		1.00	
CT	571 (50.8)	565 (47.3)	1.72 (1.40–2.12)	3.7*10^−7^	1.74 (1.42–2.14)	1.6*10^−7^
CC	344 (30.6)	273 (22.9)	2.15 (1.70–2.71)	9.1*10^−11^	2.18 (1.73–2.76)	9.5*10^−11^
CT/CC	915 (81.4)	838 (81.2)	1.86 (1.53–2.26)	4.3*10^−10^	1.89 (1.55–2.29)	4.3*10^−10^
Additive			1.46 (1.30–1.64)	1.8*10^−10^	1.47 (1.31–1.65)	6.3*10^−11^
TT/CT	780 (69.4)	921 (77.1)	1.00		1.00	
CC	344 (30.6)	273 (22.9)	1.49 (1.24–1.79)	2.0*10^−5^	1.50 (1.25–1.81)	2.3*10^−5^

CI, confidence interval; OR, odds ratio

a Adjusted for age, sex, smoking and drinking status in logistic regression models

In the stratified analysis presented in Table 2, no significant heterogeneity was found between subgroups stratified by different factors. The association between rs13361707 SNP and GCa risk was not altered by age, sex, drinking status, smoking status, and tumor site. Using 0.0038 as the significance level of Bonferroni correction for multiple testing, most results were still statistically significant.

**Table 2:** Stratification analysis for the association between PRKAA1 rs13361707 T > C polymorphism and GC risk

Variables	rs13361707 (cases/controls)	CT/CC	Crude OR 95% CI	*P*	*P*^het^	Adjusted OR ^a^ 95% CI	*P* ^a^
Median age, yr							
≤ 59	110/166	468/440	1.61 (1.22–2.11)	5.6*10^−4^	1.2*10^−1^	1.61 (1.22–2.12)	6.9*10^−4^
> 59	99/190	447/398	2.16 (1.63–2.85)	5.1*10^−8^		2.20 (1.66–2.91)	3.3*10^−8^
Sex							
Males	151/243	649/584	1.79 (1.42–2.26)	9.9*10^−7^	5.9*10^−1^	1.82 (1.44–2.30)	5.3*10^−7^
Females	58/113	266/254	2.04 (1.42–2.93)	1.1*10^−4^		2.05 (1.42–2.94)	9.5*10^−5^
Smoking status							
Never	127/192	558/416	2.03 (1.57–2.62)	5.4*10^−8^	3.4*10^−1^	2.05 (1.58–2.66)	6.6*10^−8^
Ever	82/164	357/422	1.69 (1.25–2.28)	6.0*10^−4^		1.69 (1.25–2.28)	5.9*10^−4^
Pack-year							
0	127/192	558/416	2.03 (1.57–2.62)	5.4*10^−8^	4.0*10^−1^	2.05 (1.58–2.66)	6.6*10^−8^
≤ 25 (mean)	43/110	184/243	1.94 (1.30–2.89)	1.2*10^−3^		1.87 (1.24–2.81)	3.0*10^−3^
> 25 (mean)	39/54	173/179	1.34 (0.84–2.12)	2.2*10^−1^		1.40 (0.86–2.28)	1.8*10^−1^
Drinking status							
Never	161/260	693/589	1.90 (1.52–2.38)	2.3*10^−8^	7.3*10^−1^	1.93 (1.54–2.42)	1.2*10^−8^
Ever	48/96	222/249	1.78 (1.21–2.64)	4.1*10^−3^		1.78 (1.21–2.64)	4.1*10^−3^
Tumor site							
GCA	65/356	240/838	1.57 (1.16–2.12)	3.2*10^−3^	2.2*10^−1^	1.60 (1.18–2.17)	2.5*10^−3^
NGCA	144/356	675/838	1.99 (1.60–2.48)	8.9*10^−10^		2.02 (1.62–2.51)	2.2*10^−10^

a Adjusted for age, sex, smoking and drinking status in logistic regression models

For the subsequent meta-analysis, we found four primary studies [16, 24–26]. By including the present study, a total of five studies with 9,590 cases and 9,724 controls were included in the meta-analysis. Pooled data indicated that the *PRKAA1*rs13361707 T > C SNP was strongly associated with increased GCa risk in several genetic model (CT vs. TT: OR = 1.35, 95%CI = 1.12–1.61; CC vs. TT: OR = 1.70, 95%CI = 1.34–2.16; and CT/CC vs. TT: OR = 1.44, 95%CI = 1.17–1.76, Supplementary Figure 1) without significant publication bias. Because of the notable heterogeneity across the studies, the leave-one-out sensitivity analysis was performed, and the study reported by Dong et al. [16] was considered the source of heterogeneity (dominant: I^2^, 83.1%–4.8%; homozygous: I^2^, 82.3%–0.1%; and heterozygous: I^2^, 76.1%–0.0%). More importantly, the pooled data from the final four studies still showed a strong association between rs13361707 and GCa risk, indicating the stability of the results of this meta-analysis.

## DISCUSSION

Apart from environmental and lifestyle factors for GCa risk, genetic factors in the GCa development were important in identifying at-risk populations for cancer prevention. To our knowledge, the present study was the first to demonstrate the strong association of rs13361707 SNP with an increased GCa risk in a large eastern Chinese population under five genetic models. All results consistently showed that the C allele of rs13361707 increased the risk of GCa.

PRKAA1, a subunit of the AMPK pathway, is critical to cellular activity and cancer development, and studies have demonstrated its role in cell differentiation, apoptosis, autophagy, and cancer progression [27–30] as well as in clinical prognosis [31, 32]. Recently, targeting PRKKA1 was reported as a potential way for cancer suppression [30]. Although lacking of data about the biological mechanism of the PRKKA1 pathway in GCa, the corresponding rs13361707 SNP was postulated to active the expression of PRKKA1.

The major finding in the present study is a confirmed strong association between *PRKAA1*rs13361707 in GCa risk in an eastern Chinese population under several genetic models. However, a previous GWAS study [14] confined a significant association between rs13361707 and GCa risk to the subsets of non-cardia GCa. Another replication study in Korea populations [26] indicated that rs13361707 is a risk factor for both GCA and NGCA, a consistent finding with the present study. The inconsistence of our results with the previously published GWAS study might be due to low statistical power in the analysis of subgroups stratified by tumor site. On the other hand, despite of a strong association of rs13361707 T > C polymorphism with cardia GCa or no-cardia GCa in several studies, the present study indicated a similar trend in comparison with the GWAS study by Shi et al. [13] that the rs13361707 SNP was more strongly associated with tumors in non-cardia site than tumors in cardia site. However, these results should be verified by future studies with a larger sample size.

There are some limitations in the present study. First, although age, sex, smoking status, drinking status, and tumor site were taken into consideration for subgroup analysis, other important risk factors, such as diet and HP infection, that were missing in this study, which may have also contributed to the etiology of GCa. New classification of tumor types is also important, which may have a different genetic basis in the etiology [33]. Second, the sample size of the cases in each of subgroups was largely reduced in the stratification analysis, which may have led to limited statistical power in subsequent analysis.

In conclusion, the present study confirmed that the C allele of *PRKAA1* rs13361707 was a risk factor for GCa. However, future studies should incorporate *HP* infection status and Lauren classification, which may lead to our better, comprehensive understanding of the association between the *PRKAA1* rs13361707 SNP and GCa risk.

## MATERIALS AND METHODS

### Study subjects

This study included patients who were recruited from our ongoing molecular epidemiology study of GCa, and the cases and controls were described previously [17–19]. Briefly, 1,125 unrelated ethnic Han Chinese patients with newly diagnosed and histopathologically confirmed primary GCa were recruited from Fudan University Shanghai Cancer Center (FUSCC) in Eastern China between January 2009 and March 2011. Patients other than histopathologically confirmed primary GCa were excluded. In addition, 1,196 age and sex-matched cancer-free ethnic Han Chinese controls were recruited from the Taizhou Longitudinal (TZL) study conducted at the same time period in Eastern China as described previously [20]. Blood samples of GCa patients and cancer-free controls were provided by the tissue bank of FUSCC and the TZL study, respectively. All subjects had signed a written informed consent for donating their biological samples to the tissue bank for scientific research. Demographic data and environmental exposure history of each patient were collected. The overall response rate was approximately 91% for cases and 90% for controls. This research protocol was approved by the FUSCC institutional review board.

### SNP genotyping

According to a relevant protocol, we extracted DNA from peripheral blood. The rs13361707 SNP was genotyped by the TaqMan assay with ABI7900HT real-time PCR system as reported previously [17]. Patients' status was unrevealed in the genotyping process. As recommend by the company, four negative controls (without DNA template) and two duplicated samples were included in each 384-plate for the quality control. The assays were repeated for 5% of the samples, and the results were 100% concordant.

### Statistical methods

The χ^2^ test was used to assess the differences in the distribution of demographic characteristics between cases and controls. Hardy–Weinberg equilibrium (HWE) was tested by goodness-of-fit χ^2^ to evaluate the expected distributions of genetic frequency in the controls. The association between SNP and GCa risk was assessed by odds ratio (OR) and 95% confidence intervals (CIs) in heterozygous (CT vs TT), homozygous (CC vs TT),dominant (CT+CC vs TT), recessive (CC vs CT+TT), respectively. OR values were calculated by univariate and multivariate logistic regression models. Moreover, logistic regression tests for each genetic model were adjusted for age, sex, drinking and smoking status. Furthermore, associations between the *PRKAA1* rs13361707 SNP and GCa risk were also stratified by age, sex, smoking or drinking status, and primary tumor site. We applied Bonferroni correction for multiple testing using 0.0038 as significance level. All statistical process above was achieved by SAS software (version 9.1; SAS Institute, Cary, NC)

To validate our results, we performed a mini meta-analysis with studies searched from Medline, PubMed and Embase. After using the search terms and inclusion and exclusion criteria as described in previous studies [21, 22], all primary reports were carefully reviewed, and the relevant references in these papers were also searched and reviewed by two independent authors. Then, data were retrieved from the reported studies and pooled crude ORs for heterozygous, homozygous, and dominant models were calculated. Heterogeneity between studies was estimated by Chi-square-based Q test. Pooled Ors were calculated by a fixed-effects model or random-effects model, depending on the heterogeneity between searched studies [23]. To validate the stability of the pooled results and to identify the sources of heterogeneity, the leave-one-out sensitive analysis was performed. Publication bias was shown by the funnel plot, in which the asymmetry will be estimated by Egger's liner regression test, where the statistically significant publication bias was tested out, when *P* < 0.05 determined by the *t* test as suggested by Egger. All statistical process was achieved by STATA version 10.0 (Stata Corporation, College Station, TX).

## SUPPLEMENTARY MATERIALS AND FIGURE


