# Supplementary material for: Genetic variant of PRKAA1 and gastric cancer risk in an eastern Chinese population
Source: Oncotarget. 2015 Oct 15;6(40):42661–6. doi: 10.18632/oncotarget.6124 (PMC4767461; doi:10.18632/oncotarget.6124)
Supplement: Supplementary file 1 [file oncotarget-06-42661-s001.pdf]

Genetic variant of *PRKAA1* and gastric cancer risk in an eastern Chinese population

Supplementary Materials

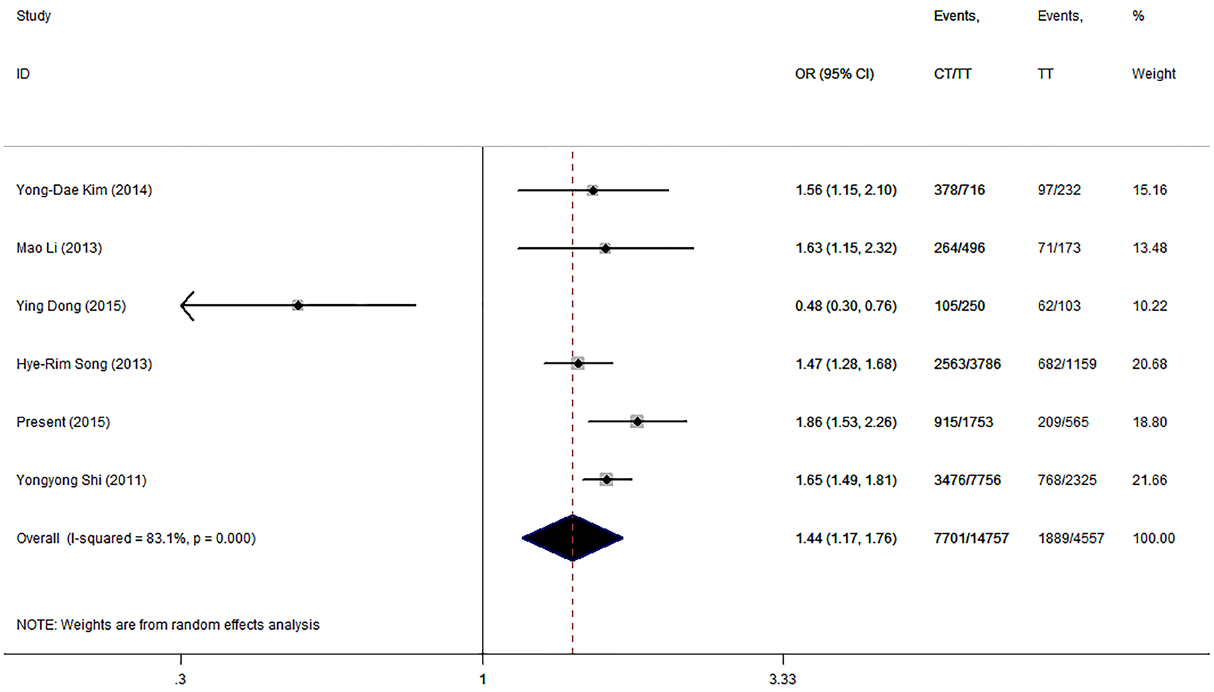

Supplementary Figure S1. Meta-analysis for the association between PRKAA1 rs13361707 SNP and GCa risk in the dominant genetic model.
